# Relationship among Activities of Daily Living, Nutritional Status, and 90 Day Readmission in Elderly Patients with Heart Failure

**DOI:** 10.3390/ijerph16245068

**Published:** 2019-12-12

**Authors:** Masahiro Kitamura, Kazuhiro P. Izawa, Masakazu Yaekura, Yumi Mimura, Yuichi Ikeda, Hitomi Nagashima, Peter H. Brubaker

**Affiliations:** 1 Department of Physical Therapy, Kokura Rehabilitation College, Kitakyushu, Kitakyushu 800-0206, Japan; pt_masa0808@yahoo.co.jp; 2 Department of Public Health, Graduate School of Health Sciences, Kobe University, Kobe 654-0142, Japan; 3 Cardiovascular stroke Renal Project (CRP), Institute, Kobe 654-0142, Japan; brubaker@wfu.edu; 4 Faculty of Sport Sciences, Waseda University, Tokorozawa 359-1192, Japan; 5 Department of Rehabilitation, Shinyukuhashi Hospital, Yukuhashi 824-0026, Japan; masa022088@gmail.com (M.Y.); momo-ysh@chime.ocn.ne.jp (Y.M.); pt-ikeda@waltz.ocn.ne.jp (Y.I.); riha@shinyukuhashihospital.or.jp (H.N.); 6 Department of Health and Exercise Science, Wake Forest University, Winston-Salem, NC 27109, USA

**Keywords:** activities of daily living, nutritional, readmission, elderly, heart failure

## Abstract

Purpose: This investigation aimed to examine the relationship among activities of daily living (ADL), nutritional status and 90-day hospital readmission in elderly heart failure (HF) patients. Methods: Participants were selected from 634 HF patients consecutively hospitalized at one institution. We investigated patient characteristics, ADL (motor and cognitive items of Functional Independence Measure (FIM)) and nutritional status (Geriatric Nutritional Risk Index (GNRI)). Data were analyzed using unpaired *t*-test, χ^2^ test, Cox proportional hazard model, and Kaplan-Meier method. Results: The 169 participants that met inclusion criteria were divided into two groups based on hospital readmission within 90 days of discharge. Body mass index (BMI) (*p* = 0.03), hemoglobin (*p* = 0.047), GNRI (*p* = 0.02) and motor-FIM (*p* = 0.007) were significantly different between the readmission (*n* = 31) and non-readmission (*n* = 138) groups. After Cox proportional hazard model analysis, GNRI (HR: 0.96; *p* = 0.048) and motor-FIM (HR: 0.97; *p* = 0.03) scores remained statistically significant. Participants were then classified into four groups based on a previous study’s cut-off values of prognosis for GNRI and motor-FIM. Readmission avoidance rate was significantly lower (*p* = 0.002) in the group with GNRI <92 and motor FIM <75. Conclusions: This study showed that motor-FIM and GNRI scores for hospitalized elderly HF patients were predictors of readmission within 90 days of discharge.

## 1. Introduction

The numbers of elderly patients with heart failure (HF) are increasing worldwide [1,2], particularly in Japan [3]. HF patients have high mortality and readmission rates resulting in dramatic increases in medical costs [4,5,6]. Short-term hospital readmission (<90 days) of HF patients is as high as 20 to 30% [7,8] and is driven by a variety of causes such as respiratory disease, orthopedic conditions, and diabetes [9,10].

Activities of daily living (ADL) define basic motor abilities (self-care, locomotion, excretion management) and the cognitive ability necessary for people to live [11]. Locomotion causes a high load compared to self-care and excretion management and is likely to be a factor causing difficulty in ADL [12]. In HF patients, ADL and nutritional status are known to be prognostic factors of long-term mortality and hospital readmission [12,13,14]. Although HF patients with poor nutritional status have been shown to have low ADL [15], no studies have examined the relationship between ADL, nutritional status and short-term hospital readmission rates.

We hypothesized that ADL and nutritional status would be independent predictors of hospital readmission and that patients with low ADL and nutritional index scores would have higher rates of 90-day hospital readmission compared to patients with high ADL and high nutritional scores.

## 2. Materials and Methods

### 2.1. Study Design and Subjects

**Figure 1:**
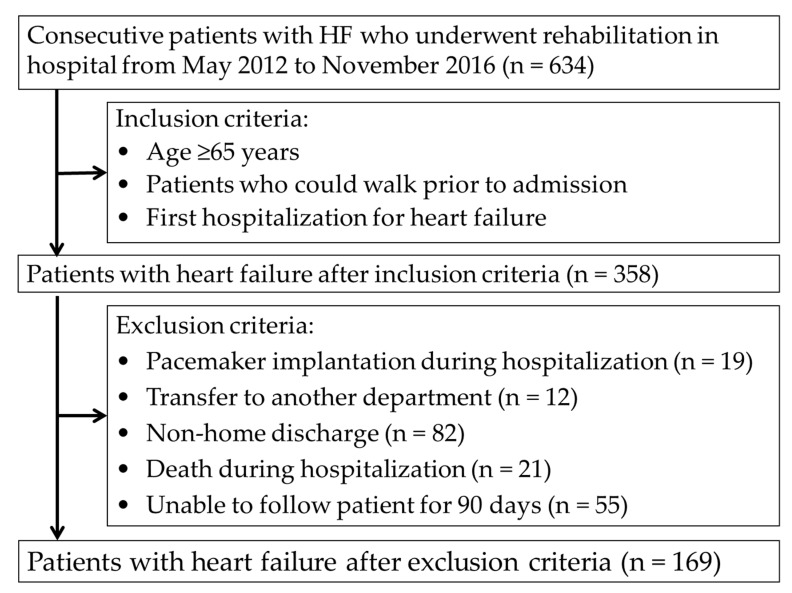
Patient flow.

This was a retrospective study in which the participants were identified from 634 consecutive HF patients who underwent rehabilitation at one acute-care hospital from May 2012 to November 2016. As shown in Figure 1, we included patients who were aged ≥65 years old, who could walk prior to admission, and who were experiencing their first hospitalization for HF. Exclusion criteria included pacemaker implantation during hospitalization, transfer to other departments, non-hospital discharge, death during hospitalization, and difficulty in following over the 90-day follow-up period. The Kokura Rehabilitation College Ethics Committee approved this study (approval no. 29-0302), and informed consent was obtained from each patient.

### 2.2. Investigation

Patient characteristics and clinical parameters obtained included age, sex, body mass index (BMI), left ventricular ejection fraction, brain natriuretic peptide, New York Heart Association class, creatinine, estimated glomerular filtration rate, hemoglobin, albumin, Geriatric Nutritional Risk Index (GNRI) score, comorbidities, medications, motor Functional Independence Measure (FIM) score, cognitive FIM score, initiation of walking exercise and length of hospital stay. Patient characteristics and clinical parameters were evaluated by retrospectively reviewing medical records. We divided the patients into two groups, the non-readmission group and readmission within 90 days group, on the basis of cut-off values of the GNRI and motor FIM scores as determined in a previous study [7].

### 2.3. Assessment of ADL

We evaluated ADL with the FIM at patient discharge from hospital [16]. The FIM is known to have high reliability [11] and validity [17], and the FIM score in heart disease is reported to be related to quality of life (QOL) [18]. The FIM was developed to assess rehabilitative aspects of patients with disabilities and consists of two domains, motor and cognitive [11]. There are 13 items in the motor domain (motor FIM): eating, grooming, bathing, dressing upper body, dressing lower body, toileting, bladder and bowel management, transfer to bed, chair, or wheelchair, transfer to toilet, transfer to tub or shower, walking/wheelchair, and stairs. There are 5 items in the cognitive domain (cognitive FIM): comprehension, expression, social interaction, problem solving, and memory. The FIM is scored over a range of 1 to 7 for each item: 1 point for total assistance, 2 points for maximal assistance, 3 points for moderate assistance, 4 points for minimal contact assistance, 5 points for supervision, 6 points for modified independence, and 7 points for complete independence. Motor FIM scores range from 13 to 91 points, and cognitive FIM scores range from 5 to 35 points. The measurements motor FIM and cognitive FIM were made by two physical therapists.

### 2.4. Assessment of Nutritional Status

The GNRI as an index of nutritional status is reported to be particularly useful in the nutritional assessment of the elderly [19] and is associated with prognosis in heart disease [20]. The baseline GNRI was calculated from serum albumin and BMI using the following formula [21]: GNRI = 14.89 × serum albumin (g/dL) + 41.7 × present body weight/[height^2^ (m^2^) × 22] = 14.89 × serum albumin (g/dL) + 41.7 × BMI/22. The patients were then divided into two groups, the low GNRI (<92) group and high GNRI (≥92) group, on basis of a previous study [15,20].

### 2.5. Assessment of Initiation of Walking Exercise

The patients included in this study followed a rehabilitation program that included the following exercises: sitting, standing, walking and ADL in accordance with the Japanese guidelines [22]. Initiation of walking exercise was defined as the day when patients with stable hemodynamics started walking exercise. Walking exercise was begun with the aid of a physical therapist after the therapist first confirmed with the patient’s physician that the patient’s hemodynamics were stable and the patient experienced no symptoms due to mild activity.

### 2.6. Assessment of Follow-up

Patients enrolled in this study were followed for 90 days after hospital discharge, with the first follow-up clinic visit scheduled within the first two weeks after discharge. Information on patient hospital readmission was obtained from the medical records by two physical therapists. This information included the date of readmission, the number of days from discharge to readmission and the causes of readmission. All-cause hospitalization within 90 days after discharge excluding hospitalization for examination was defined as readmission.

### 2.7. Statistical Analysis

Patient characteristics and clinical parameters are reported as percentages for categorical variables and mean ± SD for continuous variables. The unpaired *t*-test and chi-square test were used to compare patient characteristics and clinical parameters between the two groups. A Cox proportional hazard model for readmission within 90 days was used to ascertain whether ADL and GNRI scores were independent predictors of readmission within 90 days (hazard ratio [HR] and 95% confidence interval [CI]). The participants were classified into four groups according to the cut-off values determined in a previous study related to HF prognosis: Group 1 = motor FIM ≥75 and GNRI ≥92, Group 2 = motor FIM <75 and GNRI ≥92, Group 3 = motor FIM ≥75 and GNRI <92, and Group 4 = motor FIM <75and GNRI <92 [14,23]. Kaplan-Meier curves were constructed, and log rank tests were carried out. A p value <0.05 indicated statistical significance. Statistical analyses were performed with IBM SPSS 25.0 J statistical software (IBM SPSS Japan, Inc., Tokyo, Japan).

## 3. Results

### 3.1. Patient Flow

Participant flow in the study is shown in Figure 1. Of the 634 consecutive HF patients who underwent rehabilitation, 358 patients who met the inclusion criteria were included in this study; however, 188 patients were later excluded because of pacemaker surgery during hospitalization (*n* = 19), transfer to other departments (*n* = 12), non-home discharge (*n* = 82), death during hospitalization (*n* = 21) or difficulty in following up over 90 days (*n* = 55). Therefore, 169 patients were included in the analyses and were divided into the hospital readmission group (*n* = 31) and non-readmission group (*n* = 138).

### 3.2. Patient Characteristics

Table 1 shows a comparison of patient characteristics of the elderly hospitalized HF patients between the readmission group (*n* = 31) and the non-readmission group (*n* = 138). Compared to the non-readmission group, the readmission group had a significantly lower BMI (*p* = 0.03), hemoglobin level (*p* = 0.047) and GNRI (*p* = 0.02) and FIM scores (*p* = 0.007). The initiation of walking exercise was not significantly different between the two groups.

### 3.3. Hospital Readmission

The results of the Cox proportional hazard models presented in Table 2 show the associations between each parameter and hospital readmission within 90 days. In the univariate Cox proportional hazard model with BMI, hemoglobin, GNRI and motor FIM as covariates, BMI, GNRI and motor FIM were all independent predictors of hospital readmission. In the multivariate Cox proportional hazard model with BMI, hemoglobin, GNRI and motor FIM as covariates, GNRI (HR: 0.96; 95% CI: 0.93–0.99) and motor FIM (HR: 0.97; 95% CI: 0.94–0.99) were the independent predictors of hospital readmission within 90 day.

### 3.4. Readmission Rates by Motor FIM and GNRI

**Figure 2:**
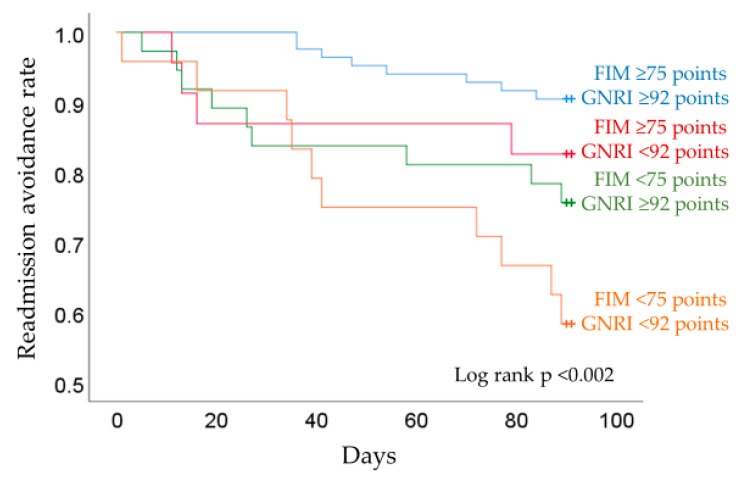
Readmission rates according to the FIM and GNRI.

In the Kaplan-Meier analysis, after dividing patients into the four groups based on cut-off values determined in previous studies [14,23], group 4 with a motor FIM score <75 points and GNRI <92 points had significantly lower rates of hospital readmission avoidance (log-rank test, *p* < 0.002) compared with the other three groups (Figure 2). In each group, the readmission avoidance rate was 90.6% for Group 1, 82.6% for Group 2, 75.7% for Group 3, and 58.3% for Group 4.

## 4. Discussion

To our knowledge, this is first study to report an association among differences in motor ADL and nutritional status and rates of short-term hospital readmission in elderly patients hospitalized for HF.

### 4.1. Characteristics of Readmission Group of Elderly HF Patients 

The rate of hospital readmission within 90 days for the HF patients in this study was 18.3%, which is slightly less than the 20–30% reported in previous investigations [7,24]. In terms of BNP and length of hospital stay reported as prognostic factors, [25,26], there was no significant difference between the two groups. The inclusion criteria of being able to walk and first hospitalization for HF and the exclusion criterion of non-home discharge might have reduced the number of severe cases and affected the findings in this study. However, the patients in the readmission group had significantly lower BMI, hemoglobin level, GNRI and motor FIM compared with those in the non-readmission group. Hospitalized HF patients with low BMI are reported to have a high rate of readmission within 30 days [27], whereas obese patients with a high BMI have a low rate of readmission [28]. Anemia in HF patients is another possible predictor of readmission within 90 days [7,24]. In addition, low ADL and a low GNRI score were reported as features of a readmission group with heart disease [19,23]. Taken together, although the subjects of the present study had a relatively low rate of hospital readmission, they did show several characteristics related to readmission within 90 days.

### 4.2. Motor ADL and GNRI as Factors of Hospital Readmission

The use of ADL and nutritional status as predictors of hospital readmission has not been investigated. The Cox proportional hazard model in the present study identified these factors as predictors of hospital readmission. Low motor FIM scores (<75 points) at discharge in hospitalized elderly patients with HF was reported to be a predictor of readmission within 90 days in a previous study [23]. Also, the motor FIM score at discharge in hospitalized HF patients undergoing rehabilitation is reported to be 60 to 70 points, and the total FIM score is 90 to 100 points [29,30]. From these reports, among hospitalized HF patients at discharge, there are many patients whose motor FIM score at discharge is lower than the readmission cutoff point. Physical function in HF patients may be reduced after discharge and can result in hospital readmission due to events such as worsening of acute heart failure, respiratory disease, orthopedic disease and diabetes or falls [31,32]. Thus, hospitalized HF patients with low ADL may have an increased possibility of hospital readmission due to the increase in cardiac load required in daily life after discharge.

The GNRI (<92 points) is a known predictor of death and readmission of hospitalized HF patients [14,20] and of death and complications of hospitalized elderly people [19]. Also, a GNRI score of less than the median (<107 points) in hospitalized HF patients has been reported as a predictor of hospital readmission [33]. Further, HF patients with poor nutritional status can experience an increased cardiac load due to fluid retention [34] and increased risk of infection due to decreased immune function [35].

### 4.3. Relation Between ADL, GNRI and the Rate of Readmission

The results of the Kaplan-Meier analysis showed that the group with GNRI <92 and motor FIM <75 points had the highest risk for hospital readmission. The main finding of this study was that the patients with low ADL and poor nutritional status had a high rate of readmission. From previous studies, ADL and nutrition of elderly HF patients are known independent factors of readmission [9,14]. However, the relation between ADL and nutrition has been only partially reported [36], and its causal relationship is unknown. In contrast, some studies reported that ADL in hospitalized HF patients with poor nutritional status is reduced, perhaps due to a decrease in muscle mass and function [37,38]. In addition, a relation between poor nutritional status and sarcopenia in heart disease and chronic disease has been reported [39,40,41]. Therefore, the relation between low ADL and poor nutritional status in hospitalized HF patients may affect their rate of readmission. Previous studies have reported that early mobilization and outpatient rehabilitation are effective in improving readmission and mobility [42,43], and continuing nutritional management from admission to after discharge is effective in improving prognosis [44]. To prevent short-term readmissions, early detection of low ADL and poor nutritional status in HF patients and mobility and nutrition interventions to improve patient outcomes are vital.

### 4.4. Strengths and Limitations of this Investigation 

This is the first study, to our knowledge, to show that motor FIM and GNRI were independent factors predictive of 90-day hospital readmission of elderly patients with HF. Although untested, improvement of motor FIM and GNRI during hospitalization may reduce the rates of readmission in these patients.

This is a single-center retrospective study with a relatively small sample size, and ejection fraction (EF) was not considered by dividing it into three types: HF with reduced EF, HF with mid-range EF and HF with preserved EF. We did not consider sex and AHA classification or patient use of sacubitril/valsartan, and ADL and nutrition information were not investigated during the follow-up period. Further, the number of severe cases may have decreased due to the patient criteria we used. There were also a significant number of patients (%) who were lost to follow-up, which may have affected the results. Finally, we did not investigate physical function such as muscle strength, body composition such as muscle quantity or quality, or the causal relationship between ADL and nutritional status in these elderly HF patients.

## 5. Conclusions

The present study found a significant relationship, both independently and in combination, between motor FIM and GNRI scores and short-term hospital readmission in elderly HF patients. Thus, it is imperative that clinicians assess and intervene to improve these measures to prevent short-term hospital readmission in these patients.

## Figures and Tables

**Table 1:** Patients’ characteristics.

Clinical Characteristics	Readmission *n* = 31	Non-Readmission *n* = 138	F or χ^2^ Value	*p* Value
Age, years	83.4 ± 5.4	80.7 ± 7.1	1.9 ^a^	0.051
Male Sex, %	58.1	51.4	0.4	0.51
BMI, kg/m^2^	21.3 ± 3.4	22.7 ± 3.3	0.1	0.03
Clinical parameters				
LVEF, %	49.1 ± 14.0	46.1 ± 16.3	2.7 ^a^	0.36
BNP, pg/mL	774.3 ± 428.7	929.6 ± 967.4	5.3 ^a^	0.17
NYHA class I/II, %	0/13/42/45	2/13/45/40	0.9	0.83
Creatinine level, mg/dL	1.8 ± 1.5	1.3 ± 1.2	6.3 ^a^	0.14
eGFR, mL/min/1.73 m^2^	41.4 ± 22.8	47.8 ± 21.3	0.2 ^a^	0.14
Hemoglobin level, g/dL	10.6 ± 2.6	11.6 ± 2.5	0.0 ^a^	0.047
Albumin level at discharge, g/dL	3.6 ± 0.5	3.7 ± 0.4	0.1 ^a^	0.17
GNRI	94.1 ± 9.7	98.6 ± 9.5	0.0 ^a^	0.02
Comorbidity, %				
Hypertension	83.9	87.0	0.2	0.65
Diabetes	25.8	37.7	1.6	0.21
Ischemic heart disease	48.4	44.2	0.2	0.67
Valvular disease	38.7	29.7	1.0	0.33
Atrial fibrillation	30.0	14.6	1.3	0.25
Orthopedic Conditions	29.0	39.1	1.1	0.29
Neurological disease	12.9	23.2	1.6	0.21
Respiratory disease	29.0	21.0	0.9	0.33
Medication				
Catecholamine	12.9	10.1	0.2	0.65
Diuretic	93.5	92.8	0.02	0.88
ACEI/ARB	51.6	40.6	1.3	0.26
β-blockers	54.8	55.1	<0.01	0.98
Rehabilitation progress				
Motor FIM score at discharge	71.2 ± 10.8	77.1 ± 10.9	0.1 ^a^	0.007
Cognitive FIM score at discharge	31.4 ± 4.8	31.2 ± 5.4	0.7 ^a^	0.87
Initiation of walking exercise, days	6.2 ± 6.3	4.9 ± 4.7	2.2 ^a^	0.20
Length of hospital stay, days	20.1 ± 11.8	17.9 ± 9.0	2.7 ^a^	0.24

Values are presented as mean ± standard deviation or %. ^a^ F value. ACEI = angiotensin-converting enzyme inhibitor; ARB = angiotensin receptor blocker; BMI = body mass index; BNP = brain natriuretic peptide; eGFR = estimated glomerular filtration rate; FIM = Functional Independence Measure; GNRI = Geriatric Nutritional Risk Index; LVEF = left ventricular ejection fraction; NYHA = New York Heart Association.

**Table 2:** Univariate analysis and multivariate analysis.

Variables	Univariate Analysis			Multivariate Analysis		
	Cox Proportional Hazard Ratio	95% CI	*p* Value	Cox Proportional Hazard Ratio	95% CI	*p* Value
BMI, kg/m^2^	0.88	0.79–0.99	0.03			
Hemoglobin level, g/dL	0.88	0.78–1.002	0.054			
GNRI	0.96	0.92–0.99	0.02	0.96	0.93–0.998	0.048
Motor FIM score at discharge	0.96	0.94–0.99	0.01	0.97	0.94–0.999	0.03

BMI = body mass index; CI = confidence interval; FIM = Functional Independence Measure; GNRI = Geriatric Nutritional Risk Index.

## References

[1] Kannel W.B., Belanger A.J. (1991). Epidemiology of heart failure. Am. Heart J..

[2] Roger V.L., Go A.S., Lloyd-Jones D.M., Adams R.J., Berry J.D., Brown T.M., Carnethon M.R., Dai S., de Simone G., Ford E.S. (2011). Heart Disease and Stroke Statistics-2011 Update: A report from the American Heart Association. Circulation.

[3] Yasuda S., Nakao K., Nishimura K., Miyamoto Y., Sumita Y., Shishido T., Anzai T., Tsutsui H., Ito H., Komuro I. (2016). The current status of cardiovascular medicine in Japan-Analysis of a large number of health records from a nationwide claim-based database, JROAD-DPC. Circ. J..

[4] Ponikowski P., Voors A.A., Anker S.D., Bueno H., Cleland J.G.F., Coats A.J.S., Falk V., González-Juanatey J.R., Harjola V.P., Jankowska E.A. (2016). 2016 ESC Guidelines for the diagnosis and treatment of acute and chronic heart failure: The Task Force for the diagnosis and treatment of acute and chronic heart failure of the European Society of Cardiology (ESC) Developed with the special contribution of the Heart Failure Association (HFA) of the ESC. Eur. Heart J..

[5] Hamaguchi S., Kinugawa S., Goto D., Tsuchihashi-Makaya M., Yokota T., Yamada S., Yokoshiki H., Takeshita A., Tsutsui H. (2011). Predictors of long-term adverse outcomes in elderly patients over 80 years hospitalized with heart failure.-A report from the Japanese Cardiac Registry of Heart Failure in Cardiology (JCARE-CARD)-. Circ. J..

[6] Sakata Y., Shimokawa H. (2013). Epidemiology of heart failure in Asia. Circ. J..

[7] Saito M., Negishi K., Marwick T.H. (2016). Meta-analysis of risks for short-term readmission in patients with heart failure. Am. J. Cardiol..

[8] Tsuchihashi-Makaya M., Hamaguchi S., Kinugawa S., Yokota T., Goto D., Yokoshiki H., Kato N., Takeshita A., Tsutsui H. (2009). Characteristics and outcomes of hospitalized patients with heart failure and reduced vs preserved ejection fraction. Report from the Japanese Cardiac Registry of Heart Failure in Cardiology (JCARE-CARD). Circ. J..

[9] Dunlay S.M., Redfield M.M., Weston S.A., Therneau T.M., Hall Long K., Shah N.D., Roger V.L. (2009). Hospitalizations after heart failure diagnosis a community perspective. J. Am. Coll. Cardiol..

[10] Dharmarajan K., Hsieh A.F., Lin Z., Bueno H., Ross J.S., Horwitz L., Barreto-Filho J.A., Kim N., Suter L.G., Bernheim S.M. (2013). Hospital readmission performance and patterns of readmission: Retrospective cohort study of Medicare admissions. BMJ.

[11] Ottenbacher K.J., Hsu Y., Granger C.V., Fiedler R.C. (1996). The reliability of the Functional Independence Measure: A quantitative review. Arch. Phys. Med. Rehabil..

[12] Dunlay S.M., Manemann S.M., Chamberlain A.M., Cheville A.L., Jiang R., Weston S.A., Roger V.L. (2015). Activities of daily living and outcomes in heart failure. Circ. Heart Fail..

[13] Tsutsui H., Tsuchihashi-Makaya M., Kinugawa S., Goto D., Takeshita A. (2006). Clinical characteristics and outcome of hospitalized patients with heart failure in Japan. Circ. J..

[14] Agra Bermejo R.M., González Ferreiro R., Varela Román A., Gómez Otero I., Kreidieh O., Conde Sabarís P., Rodríguez-Mañero M., Moure González M., Seoane Blanco A., Virgós Lamela A. (2017). Nutritional status is related to heart failure severity and hospital readmissions in acute heart failure. Int. J. Cardiol..

[15] Kinugasa Y., Kato M., Sugihara S., Hirai M., Yamada K., Yanagihara K., Yamamoto K. (2013). Geriatric nutritional risk index predicts functional dependency and mortality in patients with heart failure with preserved ejection fraction. Circ. J..

[16] Aziz E.F., Javed F., Pratap B., Musat D., Nader A., Pulimi S., Alivar C.L., Herzog E., Kukin M.L. (2011). Malnutrition as assessed by nutritional risk index is associated with worse outcome in patients admitted with acute decompensated heart failure: An ACAP-HF data analysis. Heart Int..

[17] Granger C.V., Cotter A.C., Hamilton B.B., Fiedler R.C. (1993). Functional assessment scales: A study of persons after stroke. Arch. Phys. Med. Rehabil..

[18] Scattolin F.A., Diogo M.J., Colombo R.C. (2007). Correlation between instruments for measuring health-related quality of life and functional independence in elderly with heart failure. Cad. Saude Publica.

[19] Bouillanne O., Morineau G., Dupont C., Coulombel I., Vincent J.P., Nicolis I., Benazeth S., Cynober L., Aussel C. (2005). Geriatric Nutritional Risk Index: A new index for evaluating at-risk elderly medical patients. Am. J. Clin. Nutr..

[20] Narumi T., Arimoto T., Funayama A., Kadowaki S., Otaki Y., Nishiyama S., Takahashi H., Shishido T., Miyashita T., Miyamoto T. (2013). Prognostic importance of objective nutritional indexes in patients with chronic heart failure. J. Cardiol..

[21] Yamada K., Furuya R., Takita T., Maruyama Y., Yamaguchi Y., Ohkawa S., Kumagai H. (2008). Simplified nutritional screening tools for patients on maintenance hemodialysis. Am. J. Clin. Nutr..

[22] Japanese Circulation Society Joint Working Group (2014). Guidelines for rehabilitation in patients with cardiovascular disease 2012. Circ. J..

[23] Kitamura M., Izawa K.P., Taniue H., Mimura Y., Imamura K., Nagashima H., Brubaker P.H. (2017). Relationship between activities of daily living and readmission within 90 days in hospitalized elderly patients with heart failure. BioMed Res. Int..

[24] Muzzarelli S., Leibundgut G., Maeder M.T., Rickli H., Handschin R., Gutmann M., Jeker U., Buser P., Pfisterer M., Brunner-La Rocca H.P. (2010). Predictors of early readmission or death in elderly patients with heart failure. Am. Heart J..

[25] Massari F., Scicchitano P., Iacoviello M., Passantino A., Guida P., Sanasi M., Piscopo A., Romito R., Valle R., Caldarola P. (2019). Multiparametric approach to congestion for predicting long-term survival in heart failure. J. Cardiol..

[26] Massari F., Scicchitano P., Ciccone M.M., Caldarola P., Aspromonte N., Iacoviello M., Barro S., Pantano I., Valle R. (2019). Bioimpedance vector analysis predicts hospital length of stay in acute heart failure. Nutrition.

[27] Aizawa H., Imai S., Fushimi K. (2015). Factors associated with 30-day readmission of patients with heart failure from a Japanese administrative database. BMC Cardiovasc. Disord..

[28] Zapatero A., Barba R., Gonzalez N., Losa J.E., Plaza S., Canora J., Marco J. (2012). Influence of obesity and malnutrition on acute heart failure. Rev. Esp. Cardiol. (Engl. Ed.).

[29] Galloway R.V., Karmarkar A.M., Graham J.E., Tan A., Raji M., Granger C.V., Ottenbacher K.J. (2016). Hospital readmission following discharge from inpatient rehabilitation for older adults with debility. Phys. Ther..

[30] Conner D., Barnes C., Harrison-Felix C., Reznickova N. (2010). Rehabilitation outcomes in a population of nonagenarians and younger seniors with hip fracture, heart failure, or cerebral vascular accident. Arch. Phys. Med. Rehabil..

[31] Krumholz H.M. (2013). Post-hospital syndrome-an acquired, transient condition of generalized risk. N. Engl. J. Med..

[32] Rodríguez-Pascual C., Paredes-Galán E., Ferrero-Martínez A.I., Gonzalez-Guerrero J.L., Hornillos-Calvo M., Menendez-Colino R., Torres-Torres I., Vilches-Moraga A., Galán M.C., Suarez-Garcia F. (2017). The frailty syndrome is associated with adverse health outcomes in very old patients with stable heart failure: A prospective study in six Spanish hospitals. Int. J. Cardiol..

[33] Minamisawa M., Miura T., Motoki H., Ueki Y., Nishimura H., Shimizu K., Shoin W., Harada M., Mochidome T., Senda K. (2018). Geriatric Nutritional Risk Index Predicts Cardiovascular Events in Patients at Risk for Heart Failure. Circ. J..

[34] Nakayama H., Koyama S., Kuragaichi T., Shiba M., Fujiwara H., Takatsu Y., Sato Y. (2016). Prognostic value of rising serum albumin during hospitalization in patients with acute heart failure. Am. J. Cardiol..

[35] Ueda T., Kawakami R., Horii M., Sugawara Y., Matsumoto T., Okada S., Nishida T., Soeda T., Okayama S., Somekawa S. (2014). Noncardiovascular death, especially infection, is a significant cause of death in elderly patients with acutely decompensated heart failure. J. Card. Fail..

[36] Kitamura M., Izawa K.P., Yaekura M., Mimura Y., Nagashima H., Oka K. (2019). Differences in nutritional status and activities of daily living and mobility in elderly hospitalized patients with heart failure. ESC Heart Fail..

[37] Houston D.K., Nicklas B.J., Ding J., Harris T.B., Tylavsky F.A., Newman A.B., Lee J.S., Sahyoun N.R., Visser M., Kritchevsky S.B. (2008). Dietary protein intake is associated with lean mass change in older, community-dwelling adults: The Health, Aging, and Body Composition (Health ABC) Study. Am. J. Clin. Nutr..

[38] Trobec K., von Haehling S., Anker S.D., Lainscak M. (2011). Growth hormone, insulin-like growth factor 1, and insulin signaling-a pharmacological target in body wasting and cachexia. J. Cachexia Sarcopenia Muscle.

[39] Freeman L.M., Roubenoff R. (1994). The nutrition implications of cardiac cachexia. Nutr. Rev..

[40] Wakabayashi H., Sakuma K. (2014). Rehabilitation nutrition for sarcopenia with disability: A combination of both rehabilitation and nutrition care management. J. Cachexia Sarcopenia Muscle.

[41] Htun N.C., Ishikawa-Takata K., Kuroda A., Tanaka T., Kikutani T., Obuchi S.P., Hirano H., Iijima K. (2016). Screening for malnutrition in community dwelling older Japanese: Preliminary development and evaluation of the Japanese Nutritional Risk Screening Tool (NRST). J. Nutr. Health Aging.

[42] Kono Y., Izawa H., Aoyagi Y., Ishikawa A., Sugiura T., Mori E., Yanohara R., Ishiguro T., Yamada R., Okumura S. (2019). Predictive impact of early mobilization on rehospitalization for elderly Japanese heart failure patients. Heart Vessel..

[43] Davidson P.M., Cockburn J., Newton P.J., Webster J.K., Betihavas V., Howes L., Owensby D.O. (2010). Can a heart failure-specific cardiac rehabilitation program decrease hospitalizations and improve outcomes in high-risk patients?. Eur. J. Cardiovasc. Prev. Rehabil..

[44] Bonilla-Palomas J.L., Gámez-López A.L., Castillo-Domínguez J.C., Moreno-Conde M., López-Ibáñez M.C., Alhambra-Expósito R., Ramiro-Ortega E., Anguita-Sánchez M.P., Villar-Ráez A. (2016). Nutritional intervention in malnourished hospitalized patients with heart failure. Arch. Med. Res..

